# Investigation of the Dose-Enhancement Effects of Spherical and Rod-Shaped Gold Nanoparticles on the HeLa Cell Line

**DOI:** 10.31661/gmj.v9i0.1581

**Published:** 2020-08-04

**Authors:** Samad Amani, Alireza Mehdizadeh, Mohammad Mehdi Movahedi, Marzieh Keshavarz, Fereshteh Koosha

**Affiliations:** ^1^Shiraz University of Medical Sciences, Shiraz, Iran; ^2^Department of Medical Physics and Medical Engineering, School of Medicine, Shiraz University of Medical Sciences, Shiraz, Iran; ^3^Ionizing and Non-ionizing Radiation Protection Research Center (INIRPRC), Shiraz University of Medical Sciences, Shiraz, Iran; ^4^Department of Radiology Technology, Faculty of Allied Medical Sciences, Shahid Beheshti University of Medical Sciences, Tehran, Iran

**Keywords:** Nanotechnology, Radiation-Sensitizing Agents, HeLa Cell, Radiotherapy

## Abstract

**Background:**

Cervical cancer cells are known as radioresistant cells. Current treatment methods have not improved the patients’ survival efficiently; thus, new therapeutic strategies are needed to enhance the efficacy of radiotherapy. Gold nanomaterials with different shapes and sizes have been explored as radiosensitizers. The present study compared the radiosensitizing effects of gold nanorods (AuNRs) with spherical gold nanoparticles (AuNPs) on the HeLa cell line irradiated with megavoltage X-rays.

**Materials and Methods:**

The cytotoxicity of AuNRs and AuNPs on HeLa cells in the presence and absence of 6-MV X-ray was investigated using the MTT assay. For this aim, HeLa cells were incubated with and AuNPs and AuNRs at various concentrations (5, 10, and 15 µg/mL) for 6 hours. Afterward, HeLa cells were irradiated with 6-MV X-ray at a single dose of 2 Gy.

**Results:**

The results showed that the addition of AuNRs and AuNPs could enhance the radiosensitivity of HeLa cells. Both AuNRs and AuNPs showed low toxicity on HeLa cells, while AuNRs were more toxic than AuNPs at the examined concentrations. Moreover, it was found that AuNRs could enhance the radiosensitivity of HeLa cells more than spherical-shaped AuNPs.

**Conclusion:**

This study revealed that the shape of nanoparticles is an effective factor when they are used as radiosensitizing agents during radiotherapy.

## Introduction

Radiotherapy, as a primary or adjuvant treatment modality, has remarkable advantages in cancer therapy. Despite its application in 50% of cancer patients [1], its adverse effects on the surrounding healthy tissues are inevitable due to the toxicity of high-dose megavoltage X-rays, resulting in the delivery of a limited dose to the tumor site and reduction of therapeutic efficiency [2]. In order to overcome the side effects of high-dose X-rays in normal tissues and enhance tumor response to radiotherapy, researchers have promoted the application of radiosensitizer drugs and encouraged the use of high atomic number materials, such as gold nanoparticles as radiation sensitizers [3-7]. Gold nanoparticles have unique properties, such as low toxicity, biocompatibility, chemical stability, ease of synthesis and modification, selective accumulation in tumors, and acceptable physicochemical parameters [3,8]. The mechanism by which gold nanoparticles exert their radiosensitivity through kilovoltage radiation involves photoelectric absorption [9]. On the other hand, megavoltage X-rays have been used clinically in the radiotherapy of cancer patients. Radiosensitivity induced by gold nanoparticles occurs when the radiation energy is in the megavoltage range, and the Compton effect is the major interaction [10,11]. Therefore, there must be another mechanism making cancer cells sensitive to megavoltage X-rays in the presence of gold nanoparticles. It has been suggested that free radicals, reactive oxygen species (ROS) generation, and induction of oxidative stress are major chemical and biological factors, which lead to radiosensitization, DNA damage in tumor cells, irreparable damage to the cell membrane and mitochondria [12-16], and consequently dose enhancement in radiotherapy in the presence of gold nanoparticles [1,17]. Gold nanoparticles are synthesized in different shapes and sizes, with different coatings and functions, depending on their application [18,19]. Different shapes of gold, such as nanospheres, nanorods, nanoshells, nanocages, and nanocubes, have been investigated in therapeutic and imaging fields [7,9,20,21]. Gold nanospheres (AuNPs) are prepared from 1 nm to less than 100 nm by reducing chloroauric acid, which is beneficial for imaging and radiation dose enhancement [22-27]. Gold nanorods (AuNRs) are produced by reduction of the gold salt (by means of chloroauric acid) in a solution containing cetyltrimethylammonium bromide (CTAB), which is a cationic surfactant [28]. These nanorods possess unique properties, including a small diameter (typically 25-45 nm in the longest dimension) and maximum heat conversion efficacy. The existence of two absorption peaks that correspond to transverse and longitudinal resonance is also considered a specific feature of AuNRs. Therefore, irradiating nanorods in the longitudinal plasmon resonance with near-infrared (NIR) laser makes tumor cells more sensitive to photothermal damage [29-32]. Overall, AuNRs can have more cellular uptake, depending on the surface charge and type of the functional group [33-36]. Some studies have reported that both AuNPs and AuNRs can effectively sensitize tumor cells to radiation in different cancer cell lines [18]. In this study, we aimed to determine which shape of gold nanoparticles, nanorods, or nanospheres can better sensitize tumor cells to radiation. For this purpose, we synthesized gold nanorods and gold nanospheres and compared their dose enhancement on the human cervical cancer (HeLa) cell line following the exposure of the cells to 6 MV X-rays at a dose of 2Gy.

## Materials and Methods

###  Gold Nanoparticles

Spherical and rod-shaped gold nanoparticles were provided by Nanobon Company (Tehran, Iran). The synthesis methods have been previously described in the literature [30,37]. After synthesis, nanoparticles were characterized using transmission electron microscopy (TEM; LEO906, ZEISS, Germany) to determine the shape and size of both spherical and rod-shaped nanoparticles.

###  The Protocol of Cell Culture

 The human cervical cancer cell line (HeLa) was purchased from the Pasteur Institute (Tehran, Iran) and cultured in Dulbecco’s Modified Eagle Medium (DMEM; Atocel, Austria), containing 10% heated-inactivated fetal bovine serum (FBS; Biowest, France), penicillin (100 U/mL), and streptomycin (100 μg/mL; Atocel, Austria) at 37ºC in a 5% CO2 atmosphere. The cell culture media were exchanged every two days, and the cells were recovered by trypsinizing the culture media with 0.25% trypsin- 1mM EDTA resuspended in phosphate-buffered saline.

###  The Cytotoxicity of AuNPs and AuNRs

 At passage 3 of the cell culture process, 103 cells were counted by hemocytometer, seeded onto a 96-well plate, and incubated for 12 hours to adhere. Then, the cells were treated with 5, 10, and 15 µg/mL AuNRs and AuNPs separately for six hours (eight wells for each concentration). A group of HeLa cells receiving no treatment was utilized as control cells. The percentage of cell survival was assessed after the incubation period by means of the MTT assay.

###  The Cytotoxicity of AuNPs and AuNRs when Combined with Irradiation

 Nanoparticles were incubated with HeLa cells, as described in the previous section. Prior to the initiation of radiotherapy, Hela cells were rinsed with PBS three times in order to remove the extra nanoparticles from the cell culture media, and after that, fresh media were replaced. Afterward, cells were exposed to 6-MV X-ray at a dose of 2Gy by means of a Varian linear accelerator (Varian Associates Inc., CA, USA). When the experiment was finished, the MTT assay was utilized for cell viability in response to various treatments.

###  MTT Assay

Cell viability was examined in all cells exposed to different treatment procedures, utilizing the MTT assay [19,21,32,36]. When the treatment protocols were finished, the cell culture media were removed, the cells were rinsed with PBS. Next, 100 μL FBS-free culture medium, along with 10 μL of the MTT solution (5 mg/mL) was added to each well and incubated for 4 hours. When the formazan crystals were formed, the contents of the wells were discarded, and 100 μL dimethyl sulfoxide (DMSO) was added to wells to dissolve the resulting formazan crystals. At last, the optical absorbance of the wells was recorded at a wavelength of 570 nm, by means of a microplate reader (DYNEX MRX, USA). The rate of cell survival was calculated by dividing the absorbance of treated cells to the absorbance of control cells. The optical density (OD) of dissolved formazan is equivalent to the number of viable cells. Cell viability was expressed as a percentage according to the below formula:

 Cell viability (%)= [ODsample − ODmedium)/(ODcontrol – ODmedium)]×100

 All experimental procedures were performed at least in triplicate.

###  Statistical Analysis

 The analysis of the obtained data was carried out by the SPSS software version 19(IBM, US), and the values were presented as the means and standard deviation (mean ± SD). The difference between the experimental groups was analyzed by one-way analysis of variance (ANOVA), followed by Tukey’s post hoc test. The level of statistical significance was set at P<0.05.

## Results

**Figure 1:**
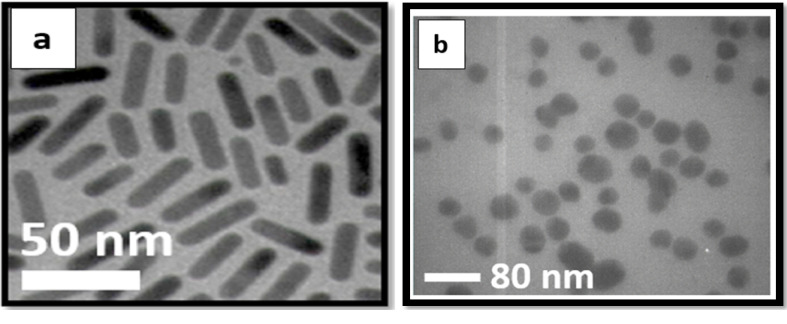
TEM micrographs of (a) gold nanorods and (b) gold nanospheres used in this study

 As depicted in Figure-1, the TEM micrographs of the prepared gold nanoparticles show that nanoparticles have spherical and rod-like shapes. An average dimension of 40nm×5nm can be observed in Figure-1(a) for AuNRs. In Figure-1(b), an average size of 40 nm is apparent for spherical AuNPs.

###  Cytotoxicity Assessment of AuNRs and AuNPs

**Figure 2:**
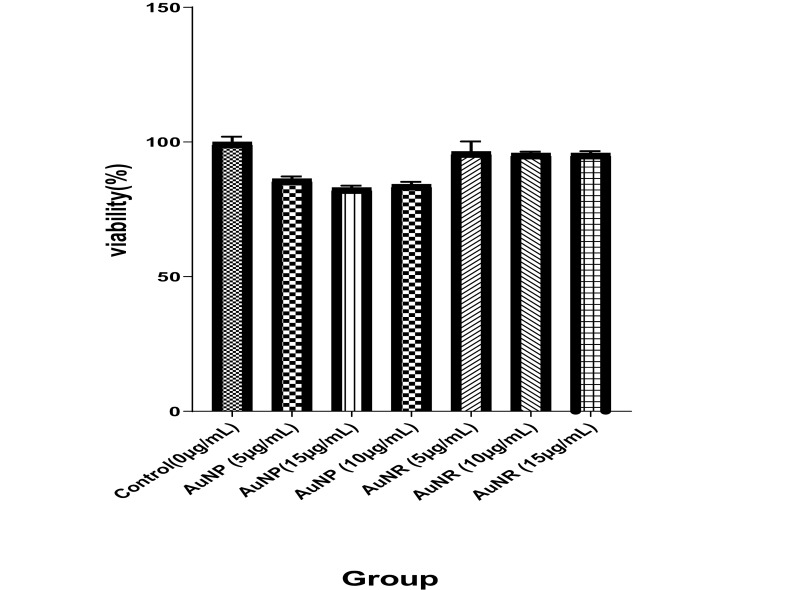
Viability of HeLa cells incubated with gold nanorods and gold nanospheres for six hours at various concentrations

 The evaluation of the cytotoxicity of formulated AuNRs and AuNPs is significant to assess the potential application of these formulations in clinical practice. The results indicated that none of the doses of AuNPs used in this study induced significant toxicity on HeLa cells in comparison with control cells. However, AuNRs caused statistically significant (P<0.05) cytotoxicity on the cells when compared with control cells. In parallel with an increase in doses of AuNPs and AuNRs, a slight difference was found in the rate of cell death; however, such a difference was not statistically (P>0.05) significant (Figure-2).

###  Effects of Radiation Therapy 

**Figure 3:**
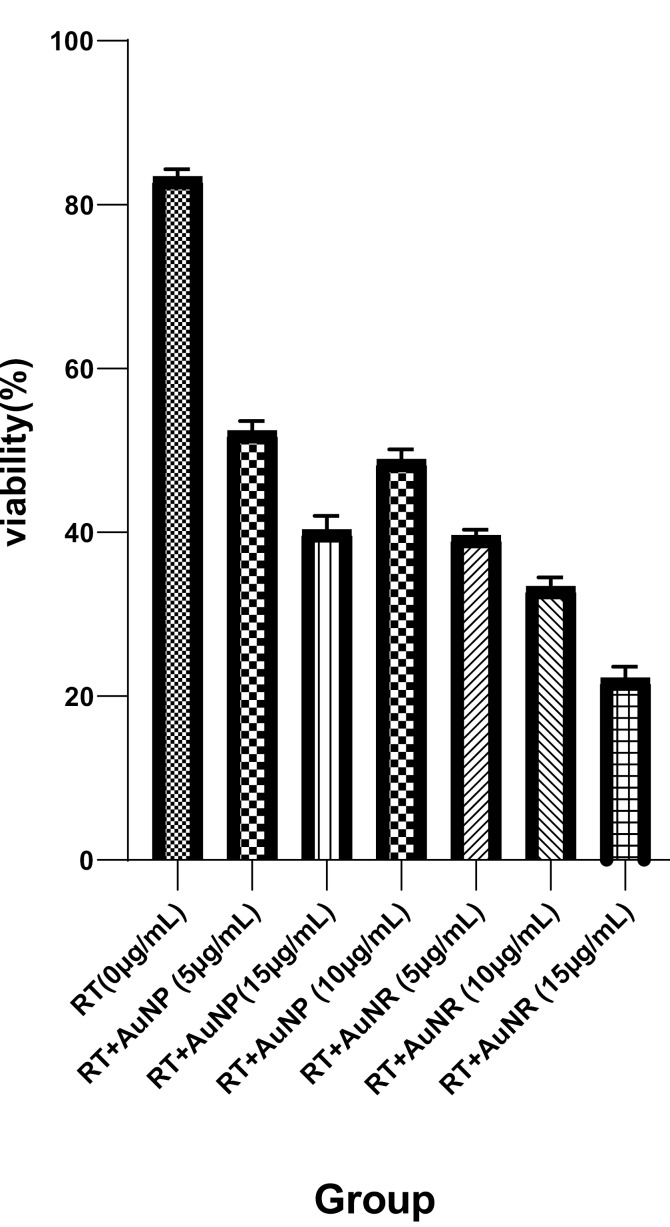
Viability of HeLa cells incubated with gold nanorods and gold nanospheres for six hours at various concentrations and irradiated with 2 Gy of 6-MV X-ray

 For the determination and comparison of the enhancement level for AuNPs and AuNRs in the process of radiotherapy with 6-MV X-rays, HeLa cells were incubated with various concentrations of both types of nanoparticles, and then, irradiated by a linear accelerator. As shown in Figure-3, both types of nanoparticles induced higher cell death in comparison with radiotherapy alone (P<0.05). A dose-dependent trend was observed for both types of nanoparticles. Cell death increased by increasing the concentration of nanoparticles. At a similar concentration, it was observed that AuNRs could induce a higher level of cell death compared to AuNPs (P<0.05). For instance, at a concentration of 15 µg/mL, cell viability was 40% for AuNPs and 22% for AuNRs.

## Discussion

Nanotechnology has been investigated as one of the new strategies for cancer treatment. The use of nanomaterials, with various sizes (1-100 nm) and shapes, has been of interest to researchers for multiple biological interfaces [8]. In particular, gold nanomaterials have applications in cancer therapy. Their positive effects as radiosensitizing agents in combination with radiotherapy have been examined in several in vitro and in vivo studies. For the first time, Haniefield *et al*. indicated the radiosensitizing effects of gold nanoparticles in 250-kVp X-ray irradiation in Murine squamous cell carcinoma in vivo [38]. According to several studies, the use of gold nanoparticles with different characteristics (e.g., shape, size, surface functionalization, concentration, and incubation period) as nano-enhancers in radiotherapy produces different dose-enhancement ratios in different cell lines [39-41]. Moreover, radiation sources with different energies may alter the radiosensitizing effect of gold nanomaterials [8]. Therefore, in the present study, the effect of the shape of gold nanoparticles (nanorods and nanospheres) in the presence of 6-MV X-ray on enhancing the therapeutic efficiency of HeLa cells was investigated. Our toxicity assessment of different concentrations of AuNRs and AuNPs showed that AuNRs are markedly more toxic than AuNPs on the HeLa cell line (Figure-1). Overall, the cytotoxicity of gold nanomaterials is due to apoptosis, necrosis, and autophagy mechanisms [42]. As displayed in Figure-2, AuNRs had a more significant impact on the dose enhancement of HeLa cells in comparison with AuNPs when treated with 6-MV X-ray at a dose of 2Gy. There are several studies, which showed the radiosensitization of gold nanoparticles with different shapes and coating materials [43,44]. In a previous study, it was shown that gold nanorods enhanced radiotherapy treatment of KB cell line in vitro. The researchers revealed that the viability of KB cells, which were treated with radiotherapy alone or 15 µg/mL of AuNRs during radiotherapy, was 81.6% and 55.1%, respectively [36]. In a study conducted by Chithrani and colleagues, they evaluated the radiosensitizing effects of 50-nm gold nanoparticles on HeLa cells and found that gold nanoparticles in combination with 6-MV X-ray enhanced radiotherapy by a factor of 1.17 [41]. In addition, Xu *et al*. applied gold nanorods on A375 melanoma cells and examined the radiosensitization effect of gold nanorods in 6-MV X-ray irradiation. They revealed that the addition of gold nanorods enhanced radiosensitivity by a factor of 1.14 due to the increase of double-stranded DNA and apoptosis in the cells [45]. As shown in Figure-2, radiosensitization could be seen in both AuNRs and AuNPs treatment groups. The mechanism of the radiosensitizing effect of gold nanoparticles in the presence of megavoltage energies cannot be described by photoelectric absorption of Au. In the presence of megavoltage energies, increasing the dose-enhancement ratio may be associated with the generation of a high level of ROS in cells, which enhances the level of oxidative stress and leads to a higher apoptosis rate [39]. Furthermore, the production of secondary electrons by MV photons and nanoparticles near DNA may cause irreparable damage to cells and enhance the effect of radiotherapy [12]. In summary, we found that AuNRs are more potent than AuNPs in enhancing the radiotherapy of HeLa cells at the examined synthesized sizes and concentrations. As cervical cancer cells are radioresistant, radiotherapy success for patients with cervical cancer depends on better dose enhancement, which can be achieved by radiosensitizers; therefore, type and shape of gold nanomaterials are among factors, which may help achieve this goal. However, further investigation of the combination of AuNRs with different sizes and coating materials must be carried out on HeLa cells.

## Conclusion

 In this study, we evaluated the effects of AuNRs and AuNPs on the radiosensitivity of HeLa cells. Our findings indicated that both AuNPs and AuNRs substantially improved the radiosensitivity of HeLa cells; however, the radiosensitization of AuNRs was remarkably higher than that of AuNPs. Therefore, it is inferred that AuNRs are useful candidates for the induction of cell death in tumor cells for patients with cervical cancer who undergo radiotherapy; however, further investigations are warranted to elucidate the precise mechanism underlying this effect.

## Acknowledgment

 The current study was supported by the Shiraz University of Medical Sciences

## Conflict of Interest

 None.
